# Phylogenetic search through partial tree mixing

**DOI:** 10.1186/1471-2105-13-S13-S8

**Published:** 2012-08-24

**Authors:** Kenneth Sundberg, Mark Clement, Quinn Snell, Dan Ventura, Michael Whiting, Keith Crandall

**Affiliations:** 1 Department of Computer Science, Utah State University, Logan UT 84322, USA; 2 Department of Computer Science, Brigham Young University, Provo, UT 84602, USA; 3 Department of Biology, Brigham Young University, Provo, UT 84602, USA

## Abstract

**Background:**

Recent advances in sequencing technology have created large data sets upon which phylogenetic inference can be performed. Current research is limited by the prohibitive time necessary to perform tree search on a reasonable number of individuals. This research develops new phylogenetic algorithms that can operate on tens of thousands of species in a reasonable amount of time through several innovative search techniques.

**Results:**

When compared to popular phylogenetic search algorithms, better trees are found much more quickly for large data sets. These algorithms are incorporated in the PSODA application available at http://dna.cs.byu.edu/psoda

**Conclusions:**

The use of Partial Tree Mixing in a partition based tree space allows the algorithm to quickly converge on near optimal tree regions. These regions can then be searched in a methodical way to determine the overall optimal phylogenetic solution.

## Background

Phylogenetic search is an NP-Hard [1] problem. It is however important to the analysis of biological sequences and the testing of evolutionary hypothesis [2]. As such it is necessary to employ heuristic methods.

A phylogenetic search begins by using a greedy heuristic to build an initial tree. This initial tree is then improved by the full search. Unfortunately, the greedy nature of the starting trees limits the effectiveness of the full search. For this reason multiple starting trees are often used, with the hope that at least one will allow the overall search to find the global minimum.

Partial Tree Mixing (PTM) addresses this issue through the use of a global representation of partition based tree space [3]. Using this representation PTM is able to quickly begin exploring this space with a global search strategy. PTM uses a strategy focused more on exploration than exploitation. By covering more of the solution space PTM leads to an increased chance of the overall search finding a global minimum. Two key features of PTM allow these goals to be accomplished. First, PTM divides a problem into smaller, more manageable subproblems, this allows for global search methods such as Tree Bisection and Reconnection (TBR) to be applied sooner. Second, PTM uses a global representation of all possible solutions, this allows for coordination between the subproblem search efforts.

### Related work

The most common heuristic method for phylogenetic search is a form of hill climbing. A given possible solution is permuted into several new solutions. The best of these solutions is in turn permuted until no better solutions are found.

The most common permutation operation is Tree Bisection and Reconnection (TBR) [4]. Common methods in current use for building an initial tree include distance based methods such as UPGMA (Unweighted Pair Group Method with Arithmetic Mean) [5] and neighbor joining [6] , as well as stepwise maximum parsimony. Both distance methods and stepwise maximum parsimony are *O*(*n*^2^) algorithms (where n is the number of taxa).

#### Distance methods

Distance methods begin by computing an all-to-all distance matrix between the taxa. This is typically the hamming distance between the DNA character sequences for each taxa though some other metrics have been used [7]. The nearest taxa are joined into a clade. Then the distance from this clade to all other taxa is computed. The method of calculating this distance varies between different distance methods. This clustering of taxa into clades continues until a complete tree has been built.

#### Stepwise maximum parsimony

Stepwise maximum parsimony begins by shuffling the taxa into a random order. The first three taxa are joined together into the only possible three taxon tree. In turn each taxon is inserted along every branch in the current tree. It is left in the most parsimonious position. This process continues until all the taxa have been added, resulting in a complete tree.

#### Tree bisection and reconnection

Tree Bisection and Reconnection (TBR) is a common means of generating new solutions during a phylogenetic search. Each iteration of TBR is an *O*(*n*^3^) algorithm and produces *O*(*n*^3^) trees to be examined. The first step is to select a branch in the tree and remove it, producing two subtrees. A branch is then selected in each of the two subtrees. A new tree is produced by reconnecting the two subtrees at the selected branches. An iteration of TBR ends when the original tree has been split along every branch and each of those splits has been rejoined in all possible ways. If one of the new trees is better, then the search continues by performing a TBR iteration on the improved tree. If no better tree is found the search ends.

### Partition based tree space

Trees can be considered as collections of bipartitions of taxa. Every branch in a tree divides the taxa into two sets. Some of these bipartitions, those arising from branches connected to the leaves, are common to all trees. These trivial bipartitions are ignored. All other possible partitions are assigned a dimension in tree space. The position of a tree is a vector whose components all have the value 1 or 0. These values respectively represent the presence or absence of the associated bipartition.

In this space there is a close relationship between the Euclidean distance between two trees and the Robinson-Foulds (RF) [8] distance between those same trees. Namely the Euclidean distance is the square root of the RF distance.

#### The hypersphere of trees

It is well known [9] that all fully resolved trees of *n* taxa have 2*n –* 3 branches. *n* of these branches are trivial, and are therefore ignored. The position of any resolved tree will therefore have exactly *n –* 3 elements with the value of 1, all others will have the value of 0. It is easy to see that the distance from this point, the position of an arbitrary fully resolved tree, and the origin is . As all such points are equidistant from the origin, it is the case that every fully resolved tree lies on the surface of a hypersphere. Unresolved trees are trees which have fewer than *n –* 3 non-trivial branches. Consider a tree lacking *m* branches, by the same argument as used for resolved trees, the distance between this tree and the origin must be , and all such trees lie on the surface of a smaller concentric hypersphere of radius .

The set of all trees, both resolved and unresolved lie upon the surfaces of a set of *n –* 3 concentric hyperspheres. At the origin lies the fully unresolved tree, which possesses no branches. The next sphere out, with a radius of 1, contains all trees with 1 branch. Each succeeding sphere contains trees with one more branch in them than the last sphere, until the final sphere of radius *n –* 3 is reached.

#### Cartographic projections

The dimensionality of tree space is *O*(*n*!!) [10], with respect to the number of taxa. Directly representing trees in this space quickly becomes prohibitive. One method of mitigating this explosive dimensionality is through cartographic projections [3]. A small number of reference vectors are chosen in tree space, these vectors need not correspond to valid trees. The coordinates of a tree are then defined as the inner products of the vector representing the tree and these reference vectors. Due to the very sparse nature of a vector which represents a tree, these inner products can be computed with a single pass over the tree in *O*(*n*) time. The method used to store the reference vectors is a hash table, and this has been shown [11] to preserve the relationship between Euclidean and RF distance.

## Results and discussion

In this section two types of results are considered. First, the work examines the effects of the parameters available to the user on the time taken and on the quality of the trees found. Second, using default settings for these parameters the method is compared with other phylogenetic search programs. PTM followed by a standard TBR search is shown to find better trees than competing methods.

### The effects of partial tree size

The PTM algorithm allows the user to set two parameters which affect the size of the partial trees during the search. The first is a maximum partial tree size. Two partial trees will not join together if the result would be a tree larger than the maximum size. The second is a minimum partial tree size. This is a soft limit, it does not prevent partial trees smaller than this limit. Rather, a tree which is at or below this minimum limit will not subdivide further.

**Figure 1:**
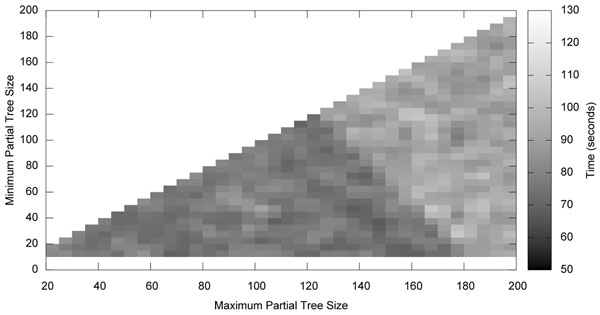
**The effects of partial tree size on time.** A graph of the time taken by the PTM algorithm as the size of the partial trees is varied. Two partial trees will not join if doing so would create a partial tree larger than the maximum size. A partial tree below the minimum size will not divide further. In general the PTM algorithm takes less time with smaller minimum and maximum sizes.

**Figure 2:**
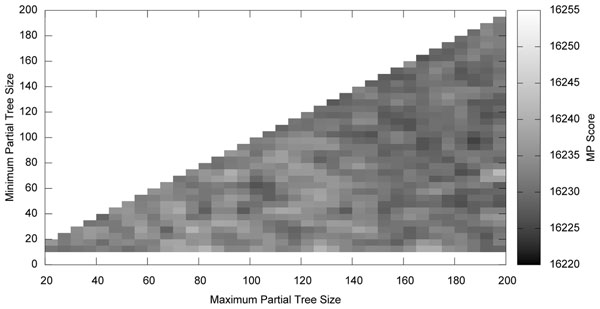
**The effects of partial tree size on score**. A graph of the maximum parsimony score of the tree found by the PTM algorithm as the size of the partial trees is varied. Two partial trees will not join if doing so would create a partial tree larger than the maximum size. Using larger partial trees tends to yield slightly better parsimony scores after PTM only, but near optimal scores are found by all searches after TBR refinement.

Figures 1 and 2 show the effects of partial tree size on time and on the score found. A PTM search was made on the Zilla data set (500 taxa) [12], setting the minimum and maximum size of the partial trees between 10 and 200 taxa. The time taken by the PTM search and the final score found were recorded. This time and score do not reflect the final tree found by the search, only the initial tree found with the PTM algorithm.

The time taken by the PTM algorithm increases as the size of the partial trees increases. Figure 1 shows this relationship. This is not unexpected as Partial Tree Mixing uses a divide and conquer strategy. There is a visible boundary between two regions of the parameter space. In one region both the minimum and maximum sizes are large and the time taken is longer. In the other region at least one of the two sizes is small.

The speed in this second region is a result of smaller tree sizes, which can be quickly optimized. As the maximum size is a hard limit it is clear how a smaller maximum size leads to smaller partial trees. It is not as obvious how a smaller minimum size leads to smaller trees. Consider a partial tree containing a small set of taxa unlike the other taxa in this partial tree. After optimization these taxa will tend to group together at the end of a long branch. This long branch will be selected as the division point when forming new partial trees. The result is a tree close to the maximum size, and a small tree. The larger tree, being close to the maximum size is less likely to join with another tree in the following iteration. Small trees do not subdivide if they are below the minimum size. If the minimum size is close to the maximum size, many of these small trees will join together to form a tree within the prescribed limit. This tends to increase the average size of the partial trees. However, a small minimum size allows these smaller partial trees to form a mix without requiring that they first join together to make large trees. This in turn tends to decrease the average size of the partial trees. The reduction in average size leads to a decrease in the time spent in the PTM algorithm.

There is little variation in the score found by PTM with respect to the size of the partial trees especially after TBR refinement. However, as shown in Figure 2, larger partial trees tend to yield slightly more parsimonious trees after PTM only. As discussed in section 5.2 larger partial trees are more accurate representations of the trees in their images. Allowing some larger trees can therefore help the quality of the final tree produced. Smaller partial trees are important for exploration of the possible space. Perhaps the best solution in terms of final tree quality is to have a large to moderate maximum tree size and a small minimum tree size. This allows a variety of both larger trees for exploitation and smaller trees for exploration.

Larger partial trees lead to better scores, but longer search times. Thus, there is a tradeoff in this parameter space between the amount of time spent by PTM and the quality of the tree found. A small or moderate minimum size is desirable for both speed and accuracy. A large maximum size increases quality while decreasing speed. The best overall results occur where the maximum size is large enough to give good results, and the minimum is small enough to compensate for this maximum size in terms of execution time. The optimal parameters likely vary by data set. This implementation uses the conservative default values of 40 and 60, respectively for the minimum and maximum sizes. While these values are likely not near the optimal for most data sets, they seem unlikely to give poor performance on any.

### Comparison with existing phylogenetic search programs

PAUP* [13] is perhaps the most widely used program for phylogenetic inference using parsimony. For this reason, the performace of PTM was compared to PAUP* using stepwise addition and TBR. TNT [14] and DCM [15] are newer programs which implement a wide variety of heuristic methods [15,16]. Partial Tree Mixing was implemented in the open source phylogenetics program PSODA [17]. These methods were tested on datasets ranging from 218 to 8780 taxa. PTM was compared against stepwise maximum parsimony where both were followed by a TBR based search until a minima was found. As the step which combines the two final partial trees is equivalent to a standard TBR search, the PTM algorithm was further refined using the Parsimony Ratchet [12] and a sectorial search [16].

The results are summarized in Table 1 and 2. Table 1 compares the results of PTM to stepwise maximum parsimony. PTM takes significantly more time than stepwise maximum parsimony. However, PTM also yields higher quality trees. Table 2 considers the effect of these higher quality trees on the overall search. This table compares the total time taken, both in PTM or stepwise maximum parsimony and in TBR. Here the value of the PTM search is made clear. The final results from PTM for all of the data sets are superior to the final results found using a stepwise tree. Furthermore, with the exception of the smallest data set, these superior trees are found in less time.

**Table 1:** PTM vs stepwise maximum parsimony

Dataset	RDPII	ZILLA	U	ARB
Taxa	218	500	6722	8780
PTM				

Score	**33534**	**16234**	**92195**	**162440**
Time	00:00:52	00:01:23	09:30:48	21:35:32

PAUP*				

Score	33934	16414	95217	165289
Difference	**+400**	**+180**	**+3022**	**+2849**
Time	<00:00:01	<00:00:0l	00:01:21	00:03:36

PAUP* (multiple trees)				
Score	33855	16386	94922	165149
Difference	**+321**	**+152**	**+2727**	**+2709**
Time	00:00:58	00:01:40	06:30:44	12:18:10

A comparison of search results between PTM and stepwise maximum parsimony on several datasets. Note that in every case PTM found more parsimonious trees, but in much more time. When stepwise maximum parsimony was used to find multiple starting trees (300), PTM still found more parsimonious trees.

**Table 2:** PTM vs PAUP*

Dataset	RDPII	ZILLA	U	ARB	PROTO
Taxa	218	500	6722	8780	25057
PTM					

Score	**33515**	**16218**	**92195**	**162438**	**810231**
Time	1:18:29	2:32:03	10:39:56	24:47:00	23:49:40

PAUP*					

Score	33565	16221	93106	162906	
Difference	**+50**	**+3**	**+911**	**+468**	
Time	0:01:28	15:42:19	20:10:42	29:13:33	

TNT					

Score	42166	16219	201259	170356	
Difference	**+8651**	**+1**	**+109064**	**+7918**	
Time	0:00:48	0:00:07	1:31:54	1:47:45	

A comparison of search results between PTM and PAUP*, TNT, and DCM on several datasets. Note that in every case PTM followed by PSSS found a more parsimonious tree than PAUP* using stepwise maximum parsimony followed by TBR. In all but the smallest case, where the overhead of PTM is more difficult to overcome, this tree was found in less time. TNT finishes much faster than PTM, but finds less parsimonious trees. DCM experienced errors in processing many of the data sets and reported no score in these cases. However, the result from the successful run was inferior. Only the PTM method was able to process the largest data set of protobacteria, containing more that 25 thousand taxa.

**Figure 3:**
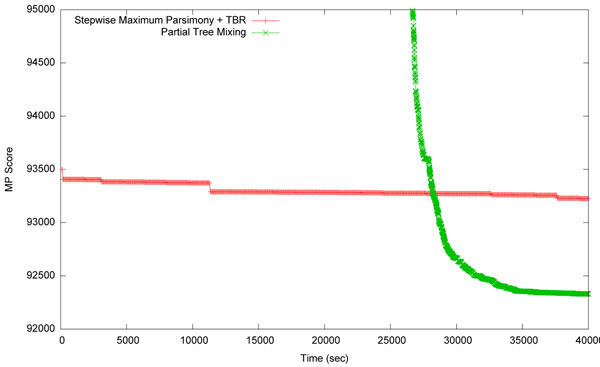
Scores found over time for PTM and PAUP*. A comparison of scores found over time between Partial Tree Mixing (PTM) and PAUP* [13] (Stepwise Maximum Parsimony followed by TBR). Although PAUP* achieves better scores during the early phases of the search, PTM achieves significantly better results after 30000 seconds.

A trace of a typical result is shown in Figure 3, the figure shows a search through a set of 6722 taxa. This trace only shows the TBR search after stepwise to the point in time when PTM returned an initial tree. The scores for the PTM search do not include any TBR refinement. For much of the search time the current tree score of PTM is poor. However, while PTM is exploring low scoring trees it is sampling from a broad area of tree space. It does this so that later phases of the search will not be caught in local minima. The value of this exploration is seen in how quickly the score improves, attaining a far superior answer in less time than traditional methods. The solution from PTM, before any TBR refinement, implies 900 fewer mutation events then the solution found by PAUP*. The solution found was then passed on to a TBR search where further improvements were made, though this is not shown in the figure.

## Conclusions

Partial Tree Mixing is a method for producing an initial phylogenetic tree for use in common hill climbing methods. Current methods produce a tree built using only local information such as pairwise distances or stepwise parsimony. As the trees produced by these greedy methods can limit the final score after a TBR search it is common practice to start many searches from different starting trees. A TBR search is much more expensive than any of the current starting methods and this duplication of effort outweighs the benefits of a quickly produced starting tree.

PTM produces a tree based on a global search of tree space guided by a partitioned based representation of all possible solutions. Although much more time is expended in producing this tree, results show that the tree produced is of better quality than a tree found using stepwise maximum parsimony followed by an equal amount of time spent in a TBR search. The exploratory nature of the PTM search greatly reduces the need for multiple searches, as PTM produces excellent starting trees. This in turn reduces the overall search time, as duplicate searches are not needed. Overall, a search started with a PTM produced tree finds better solutions in less time.

## Methods

Partial Tree Mixing (PTM) is intended to initialize a search through a data set with a large number of taxa. A concern with current methods is that they take *O*(*n*^2^) steps before any searching can occur. When *n* is small this is not problematic, especially as no prior methods have proposed any other solutions to initializing a TBR-based search. While PTM takes more than *O*(*n*^2^) steps before handing over an initial tree to a TBR-based search, it is able to begin global searching after only *O*(*n* log *n*) steps.

### Overview of partial tree mixing

Partial Tree Mixing is a divide and conquer strategy for building an initial search tree. A primary goal of PTM is to use partial trees (see Definition 6.2), containing only a subset of the taxa to search tree space. By keeping the number of taxa small, PTM is able to search faster than traditional methods.

Unlike previous methods, PTM is not a greedy heuristic. Although it employes heuristic techniques, PTM uses a representation of the global search space to insure that a large portion of the space is explored. This global representation is based on considering trees as collections of bipartitions [3]. Each bipartition is associated with a dimension. The location of a tree in this tree space is determined by which bipartitions are in the tree. As a result topologically similar trees are close together. A thorough exploration of this space therefore leads to a thorough examination of possible topologies. This reduces the necessity of finding multiple starting trees to avoid local minima.

The PTM method is based on the idea that an unresolved tree is an approximation of all the resolutions (see Definition 6.3) of that tree. This is a reasonable assumption as the unresolved tree contains the information which is common to all of its resolutions. The quality of the approximation depends on the degree of resolution of the unresolved tree. The fully unresolved tree contains no information about any of its resolutions, while the fully resolved tree contains perfect information about its resolution. However, while the quality of the approximation increases as the degree of resolution increases the number trees which are represented by the approximation decreases. PTM leaves the size of partial trees, and therefore the degree of resolution, to the user. Section 2.1 discusses the effects of varying this parameter. The region of the global tree space which contains all of these resolutions is the image (see Definition 6.6) of the unresolved tree.

During tree mixing, unresolved trees are chosen which have images covering new portions of tree space. As the partial trees are kept small, many of these exploratory searches can be accomplished in a small amount of time. Although this exploratory effort is important to the success of PTM, the partial trees are constrained to only consider improvements throughout the process.

**Figure 4:**
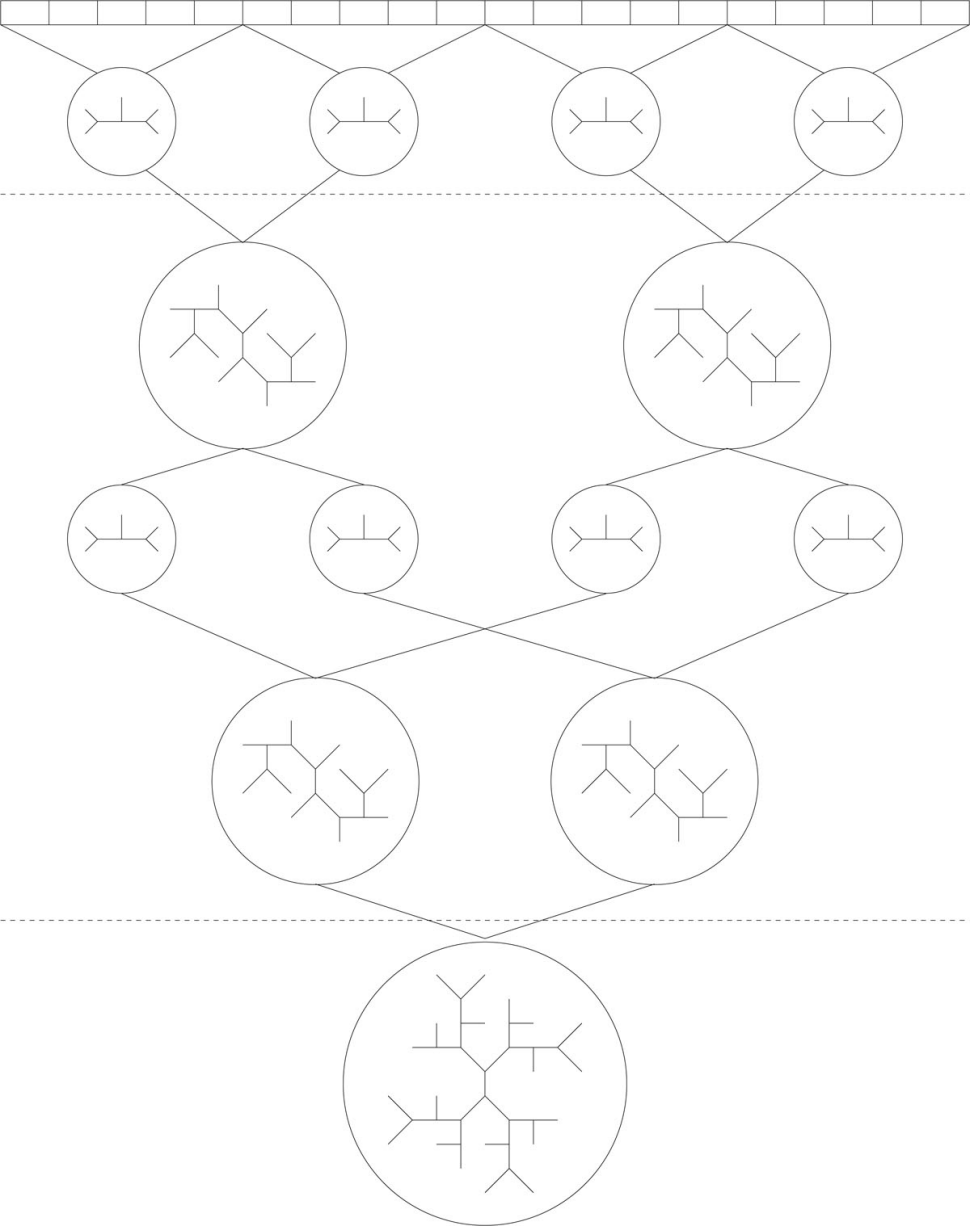
**A brief overview of the PTM algorithm**. A brief overview of the PTM algorithm. In the first phase the taxa are sorted and grouped into small disjoint sets. A stepwise maximum parsimony tree is built from each of these sets. In the second phase these trees are repeatedly joined, refined, and divided. The division of trees is identical to the tree bisection portion of the TBR algorithm. Likewise, the joining of these trees is identical to the tree reconnection portion of TBR. For this joining to work, it is essential that no taxa is represented twice. To insure this, during a PTM search all leaves on all partial trees are uniquely labeled. In the final phase no division occurs. Thus, the trees continue to grow in size until a tree containing all of the taxa is produced.

Figure 4 shows a graphical overview of the PTM process. First the taxa are divided into disjoint sets and the initial partial trees are built (Section 5.1). Then the partial trees mix together, exploring the global tree space (Section 5.2). Finally the partial trees are joined to build a fully resolved tree (Section 5.3), which can then be passed on to the usual TBR-based search.

## Algorithm

The PTM algorithm consists of three phases described in detail below. First, a set of initial partial trees is built. Next, these trees are mixed to improve their quality. Then a final complete tree is built using these partial trees. Once this tree is built it can be further refined using traditional methods.

### Initial partial trees

To begin the PTM algorithm the taxa are first divided into small disjoint subsets. An effort is made to place similar taxa into the same subset. This is done by computing a pairwise distance between an arbitrary taxon and all others. As taxa are usually given as DNA character sequences this distance is an edit distance between the two sequences. The taxa are then placed into a priority queue using this distance. Next the taxa are drawn off this queue in nearly even groups of 50-100 taxa. This *O*(*n* log *n*) method avoids the high costs of other distance methods. Then the initial partial trees are formed using stepwise maximum parsimony on these much smaller data sets. These initial trees are finally refined using TBR. As these partial trees have fewer than 100 taxa a local minima can usually be found in a few seconds. Thus the first searching of tree space occurs after *O*(*n* log *n*) steps, much sooner than under traditional methods which are *O*(*n*^2^)*.*

### Tree mixing

Once PTM has a set of disjoint locally optimal partial trees, the search progresses via tree mixing. In this process two partial trees are joined to form a new partial tree. This tree is refined with TBR to find a local minima. The optimized partial tree is then divided again into two new partial trees. These trees in turn join with others. This both keeps the size of each tree small, so that TBR is effective, and allows information to spread through the system.

Partial trees never join with their siblings from the previous division as this results in no progress. Beyond this constraint, they are free to join with any other partial tree. Partial trees remember where the tree they split from was located, and seek partners to join with that will place the new combined tree as far from the old combined tree as possible. The purpose of this preference is as a heuristic method to cover as much of the hypersphere of trees as possible with the images of the larger partial trees.

**Figure 5:**
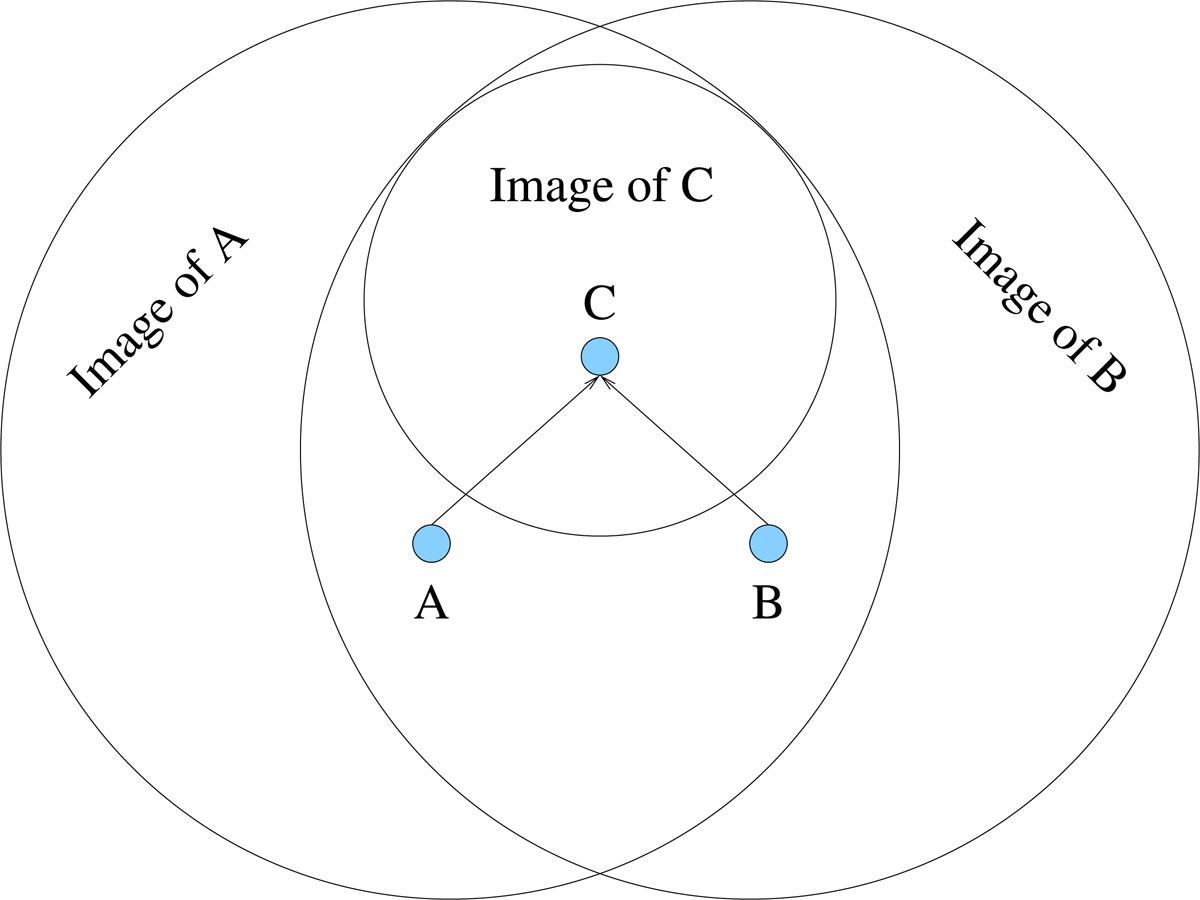
**The effects of partial tree joining**. A depiction of the effects of partial tree joining on the images of the partial trees involved. Partial trees A and B are combined to form partial tree C. A and B have fewer branches than C, therefore they can be resolved into more trees and each has a larger image than C. The image of C is contained in the intersection of the image of A and B, as any resolution of C is also a resolution of A and a resolution of B. Although the image of C is smaller it is more detailed, as C is more resolved.

The image of a joined partial tree encompasses the intersection of the images of its member trees. Figure 5 shows this relationship graphically. The larger partial tree has a smaller image than the partial trees of which it is composed. Though smaller, this image is a more accurate representation of the quality of trees in that region of tree space. It is therefore important to cover as much of tree space as possible with these higher quality images. This is accomplished by building new partial trees as far away as possible from previous partial trees. This distance helps to encourage the exploration of tree space.

It is not necessary to remember the location of old partial trees from iterations other than the immediately proceeding iteration. While the image of a partial tree contains all resolutions of that tree, it is not the case that no other trees lie within this region of tree space. It is unlikely that a partial tree whose image has a large overlap with the image of a previously considered partial tree contains no new trees. Additionally, as the search progresses the overall quality of the partial trees being used improves. It may be helpful to reexamine an area covered by an old image in light of this new information.

**Figure 6:**
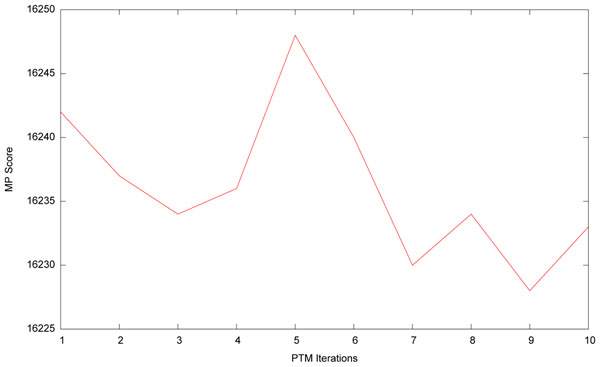
**PTM score vs PTM iterations.** A comparison of the score of a PTM tree against the number of PTM iterations used in the search.

Figure 6 shows a comparison of the number of iterations used and the score found by PTM. The spike in this graph around 5 iterations occurs as PTM finds a large local minima which TBR has a difficult time escaping. With fewer iterations this minima is not found, and with more iterations it is escaped. The optimal number of iterations is data set dependent. In this work we used three iterations which worked well across the data sets tested. This small number of iterations greatly reduces any concern of duplicating effort from prior iterations.

Partial trees are divided on their longest branch. If parsimony is the optimality criterion, this is the branch which requires the greatest number of mutation events. If likelihood is used then branch length has the usual meaning. This tends to keep taxa together during mixing that are together in an optimal tree. It also allows those taxa which are most different from others in a partial tree to migrate to a different partial tree where they can be placed more appropriately.

### Building the tree

After a prescribed number of tree mixing iterations, PTM begins to build a fully resolved tree. Partial trees continue to seek partners for joining as before. However no partial tree division occurs. Thus the partial trees become larger and larger until a fully resolved tree is built. During this phase PTM does progressively less exploration and progressively more exploitation. This tree is then passed on to a TBR based search or some other method as would be done with a stepwise maximum parsimony tree.

## Proofs and definitions

This section contains formal definitions of terms used in this work.

**Definition 6.1.** Tree: A tree is a connected acylclic graph with no vertices of degree two. A tree is **resolved** if its vertices are only of degree one or three, otherwise it is **unresolved.** The edges of this graph are also called **branches**. The vertices of degree one are called **leaves.** The leaves of a tree are labeled with taxa.

**Definition 6.2.** Partial Tree: A partial tree is a resolved tree whose leaves are labeled with a subset of the taxa.

**Definition 6.3.** Resolution of unresolved trees: A resolved tree(*R*) is a resolution of an unresolved tree (*U*) if the resolved tree can be iteratively constructed from the unresolved tree using the following operation. Select vertex *v* of at least degree four. Call the set of vertices directly connected to *v*, *G.* Remove *v* and all edges between *v* and any member of *G* from the graph. Add two new vertices *v*_1_and *v*_2_ and the edge (*v*_1_, *v*_2_) to the graph. Finally for each element *g* of *G* add either (*v*_1_, *g*) or (*v*_2_, *g*) such that *v*_1_ and *v*_2_ are at least degree 3.

**Figure 7:**
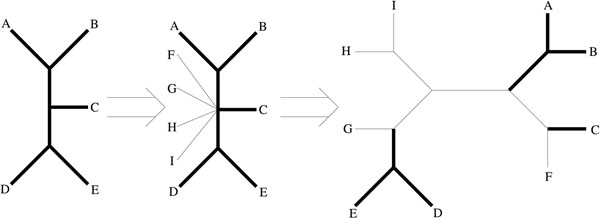
**A partial tree and resolution**. A partial tree containing only five taxa, is resolved by adding the missing taxa forming an unresolved tree nine taxa. The vertex of degree 7 is then divided as described in Definition 6.3 until a resolved tree of nine taxa has been constructed. This resolution of the first tree is contained in the image of that partial tree.

**Definition 6.4.** Resolution of partial trees: A resolved tree is a resolution of a partial tree (*T*) if it is the resolution of an unresolved tree (*U*) that can be constructed in the following manner: Let *V* be the set of vertices in *T* that are not leaves. For every taxa not in the partial tree add a vertex *t* labeled with the taxa and an edge (*t*, *v*) where *v* ∈ *V.* Figure 7 shows this process.

### Images under cartographic projections

Cartographic projections are used to build a representation of the global tree space. This section covers the properties of images of various tree constructs under this projection.

**Definition 6.5.** Properties of the Cartographic Projections:

• The projection maps branches to vectors in ℝ*^n^*

• The components of these vectors are uniformly distributed from [–1,1]

• Resolved trees are projected to the sum of the projections of their component branches, a point in ℝ*^n^*

• All trees lie in ℝ*^n^*, also referred to as global tree space

See [3] for details.

**Definition 6.6.** Image of an unresolved or partial tree: The image of an unresolved or partial tree is defined as a volume which contains the image of all resolutions of this tree.

**Theorem 6.7. ***The image of an unresolved tree is a hypersphere.*

*Proof.* Consider an unresolved tree of *n* taxa which has *n – m –* 3 branches. The location of the image of any resolution of this tree contains two components. The first is the sum of the images of the *n – m –* 3 branches from the unresolved tree. This will be the same for all resolutions, and lies at the center of the hypersphere. The second is the sum of the images of the *m* branches constructed during resolution. The magnitude of the components of these vectors is at most 1. If the projection is into *d* dimensions then the maximal magnitude of such a vector is . With *m* such vectors, the magnitude of their sum can not exceed . This is the radius of the hypersphere. The image of any resolution is the sum of the center of the hypersphere and some vector with magnitude less than or equal to the radius of the sphere. Clearly all such images are contained by this sphere.

**Theorem 6.8. ***If two unresolved trees can he constructed from the same partial tree then the centers of their images are not separated by more than**.*

*Proof* Consider a partial tree with *n – m* taxa. This tree has *n – m –* 3 branches. The branches added when resolving a partial tree to an unresolved tree are identical. Thus, two such resolutions can at most differ by *n – m –* 3 branches. If two such resolutions differed by every branch possible, and the vectors associated with the differences were all of the maximal magnitude and in opposite directions, the centers of the two images could not be separated by more than .

**Theorem 6.9. ***The image of a partial tree is bounded by a hypersphere.*

*Proof.* Consider a partial tree with *n – m* taxa. Unresolved trees which are resolutions of this tree will have *n – m –* 3 branches. The hyperspheres which contain the images of these unresolved trees will all be of radius , where *d* is the dimensionality of the image space. By Theorem 6.8 the most distant unresolved trees are not separated by more than . Thus it is clear that all trees in the image of any of the hypersphere images of the unresolved trees can be circumscribed by a single larger hypersphere of at most radius .

## Authors' contributions

KS wrote the majority of the code to implement the algorithm and ran the experimental data to get the results. MC and QS collaborated in algorithmic development and wrote supporting code for the implementation. KC and MW assisted in developing biologically relevant data sets and in providing insights into related algorithms and approaches that were investigated in determining a successful solution. DV provided feedback on how to frame the arguments in a sound manner and provided invaluable feedback on the written document.

## Competing interests

The author(s) declare that they have no competing interests.
